# Amazonian Cyanobacteria as a Source of Bioactive Compounds With Antimicrobial and Cytotoxic Properties

**DOI:** 10.1002/cbdv.202501582

**Published:** 2025-09-22

**Authors:** Samuel Cavalcante do Amaral, Luciana Pereira Xavier, Mariana Reis, Rhuana V. Médice, Janaína Morone, Raquel Silva, João Morais, Vitor Vasconcelos, Agenor Valadares Santos

**Affiliations:** ^1^ Laboratório de Biotecnologia de Enzimas e Biotransformações , Instituto de Ciências Biológicas Universidade Federal do Pará Belém Pará Brazil; ^2^ CIIMAR/CIMAR ‐ Interdisciplinary Centre of Marine and Environmental Research University of Porto Matosinhos Portugal; ^3^ Departamento de Análises Clínicas e Toxicológicas, Faculdade de Ciências Farmacêuticas Universidade de São Paulo São Paulo Brazil; ^4^ Departamento de Biologia, Faculdade de Ciências Universidade do Porto Porto Portugal

**Keywords:** bioactivity, cyanobacteria, LC–MS‐based metabolomic, OSMAC (One Strain Many Compounds), phycobiliproteins

## Abstract

Cyanobacteria are photosynthetic microorganisms known for producing diverse bioactive compounds. This study explores the bioactivity of extracts and fractions from Amazonian cyanobacteria and their pigment and phenolic compound production. A fraction from *Synechococcus* sp. CACIAM 05 showed cytotoxicity against hCMEC/D3 and MG‐63 cells. UPLC‐HRMS/MS analysis of this fraction (C5FD) identified pheophytin and phorbide derivatives as key bioactive molecules. In antimicrobial assays, another fraction from the same strain exhibited bactericidal activity against *Bacillus subtilis* ATCC 6633, with a MIC of 40 µg mL^−1^. Additional strains also showed antibacterial effects, though with higher MICs. Allelopathic activity identified eleven bioactive strains, including a synergistic effect between *Desmonostoc* sp. CACIAM 45 and *Synechocystis* sp. CACIAM 05. Aqueous extracts from *Lyngbya* sp. CACIAM 07 and *Desmonostoc* sp. CACIAM 45 showed the highest total phenolic content. Cultivation of *Desmonostoc* sp. CACIAM 45 in different media indicated CHU‐10 and ASM as optimal for phenolic and phycobiliprotein production. These findings contribute to the field by unveiling novel bioactivities and metabolic profiles of Amazonian cyanobacteria, supporting their potential as a valuable source of natural products for pharmaceutical and biotechnological applications.

## Graphical abstract

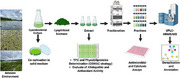

## 1 Introduction

Cyanobacteria are the oldest group of oxygenic photosynthetic organisms on the Earth, with fossil records dating back 3.5 billion years [1]. These photosynthetic microorganisms are widely distributed in freshwater, marine, and terrestrial environments [2], and are capable of surviving in extreme conditions, such as cold and hot deserts [3, 4] due to several morphological and physiological adaptations [5, 6]. The production of secondary metabolites has been one of the strategies investigated mainly by the scientific community. These natural products are chemically diverse and are known for their unique structural features [7, 8]. Furthermore, they possess a wide range of bioactivity, including anticancer, antiviral, fungicide, bactericide, and algaecide activities [8, 9].

Between 1970 and 2019, around 1630 unique molecules produced by cyanobacteria were identified, distributed across 260 families, with a predominance of peptide‐based structures [10]. The increasing number of cyanobacterial culture collections worldwide has aided in the description of new species and strains, contributing to a better understanding of the group's morphological and genetic diversity [11]. However, studies focused on the chemical composition and bioactive potential of these organisms remain limited, remaining an unexplored source with a high potential for the discovery of unknown secondary metabolites [12]. In the Amazon rainforest, this knowledge is even scarcer [13].

The Amazon region harbors the largest river basin and tropical rainforest on the planet, hosting a wide variety of ecosystems—including savannas, floodplains, igapó forests, upland forests, mountainous areas, and mangroves [14]. Cyanobacteria represent a significant part of the microbial communities in the Amazon Basin, especially the diazotrophic species, which are an essential nitrogen source in these ecosystems [15]. Furthermore, they can be a food source for benthic organisms and zooplankton populations. The systematic study of the Amazon biome has allowed the discovery of a novel genus [16, 17]. The genome of some strains harbors biosynthetic gene clusters involved with the adaptation processes, such as those that confer resistance to heavy metals and production of terpenes, bacteriocins, non‐ribosomal peptides, and polyketides, highlighting their potential as a source of novel natural products [13].

Based on the above scenario, the Amazonian Collection of Cyanobacteria and Microalgae (CACIAM) was established in 2010. This culture collection is located in the Biomolecular Technology Laboratory and Enzyme Biotechnology and Biotransformation Laboratory (Institute of Biological Sciences—UFPA—Belém, Pará, Brazil) and harbors around 80 cyanobacterial strains isolated mainly from freshwater. While some studies have explored the fatty acid profiles of these strains for biofuel production and their enzymatic activity, investigations into their bioactive potential remain scarce [18, 19].

Therefore, the present study aimed to evaluate the cytotoxicity, antimicrobial, allelopathic, and antioxidant potential of cyanobacteria from CACIAM and determine their extracts' total phenolic and phycobiliprotein (PBP) content. In addition, the bioactive compound present in the cytotoxic fraction was identified through untargeted metabolomic analysis using high‐resolution electrospray ionization mass spectrometry coupled with liquid chromatography (HPLC–HRESIMS).

## 2 Results and Discussion

### 2.1 Phylogenetic Analysis

**FIGURE 1:**
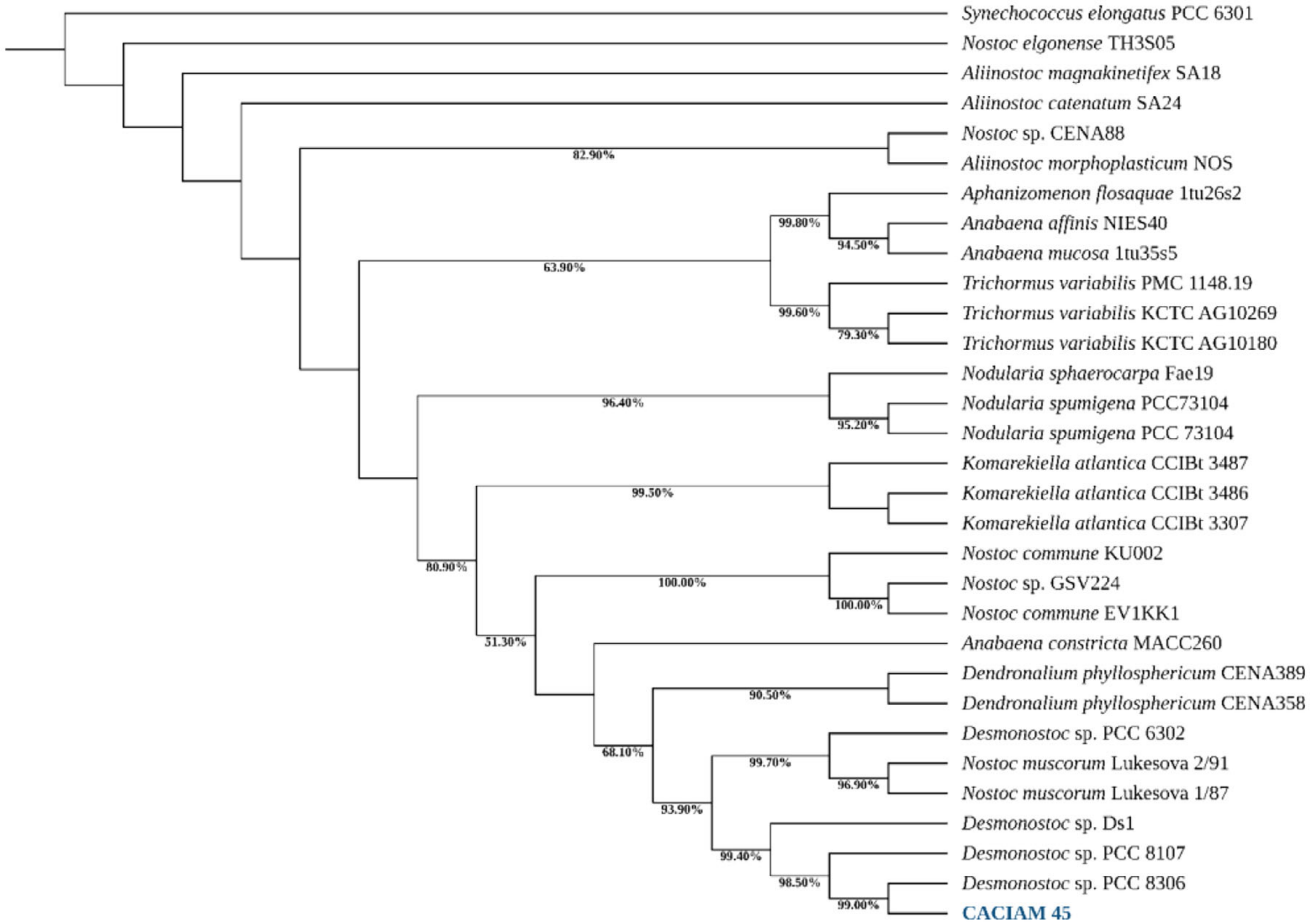
Maximum likelihood phylogenetic tree based on 31 partial 16S rRNA gene sequences of cyanobacteria. *Synechococcus elongatus* PCC 6301 was used as the outgroup. CACIAM 45 identified in this work is indicated in blue bold. Bootstrap values over 50% are indicated at the nodes.

The strain CACIAM 45 was initially classified within the order Nostocales due to its morphological similarity with the group. A phylogenetic analysis based on the 16S rRNA gene, combined with reference sequences from GenBank, revealed that CACIAM 45 and *Desmonostoc* sp. PCC 8306 are closely related within a highly supported clade (99.40% bootstrap) that also includes *Desmonostoc* sp. PCC 8107 and *Desmonostoc* sp. DS1 (Figure 1). These significant genetic and evolutionary links between these species are highlighted by this strong phylogenetic grouping, which points to a common ancestor and similar features within the genus *Desmonostoc*.

### 2.2 Cytotoxicity

The cytotoxic effects of extracts from the cyanobacteria *Desmonostoc* sp. CACIAM 45 and *Synechococcus* sp. CACIAM 66 were evaluated on HepG2 hepatocellular carcinoma cells, HCT‐116 colorectal carcinoma cells, and hCMEC/D3 brain endothelial cells using the 3‐(4,5‐dimethylthiazol‐2‐yl)‐2,5‐diphenyltetrazolium bromide (MTT) assay. Both aqueous and methanolic extracts exhibited low or negligible cytotoxicity at concentrations ranging from 12.5 to 200 µg mL^−1^, suggesting a minimal presence of cytotoxic compounds.

Investigations into novel bioactive compounds from natural sources typically begin with crude extracts, followed by activity‐guided fractionation. While this approach has contributed to the discovery of new secondary metabolites, it presents challenges due to the chemical complexity of crude extracts [20], which can result in observed bioactivity being attributed to the combined effects of multiple compounds rather than a single purified compound, potentially leading to false‐positive results. Pre‐fractionation can mitigate these issues by reducing antagonistic interactions among compounds and enhancing the activity of individual metabolites [21]. Based on this rationale, we also included fractions in our screening work.

**FIGURE 2:**
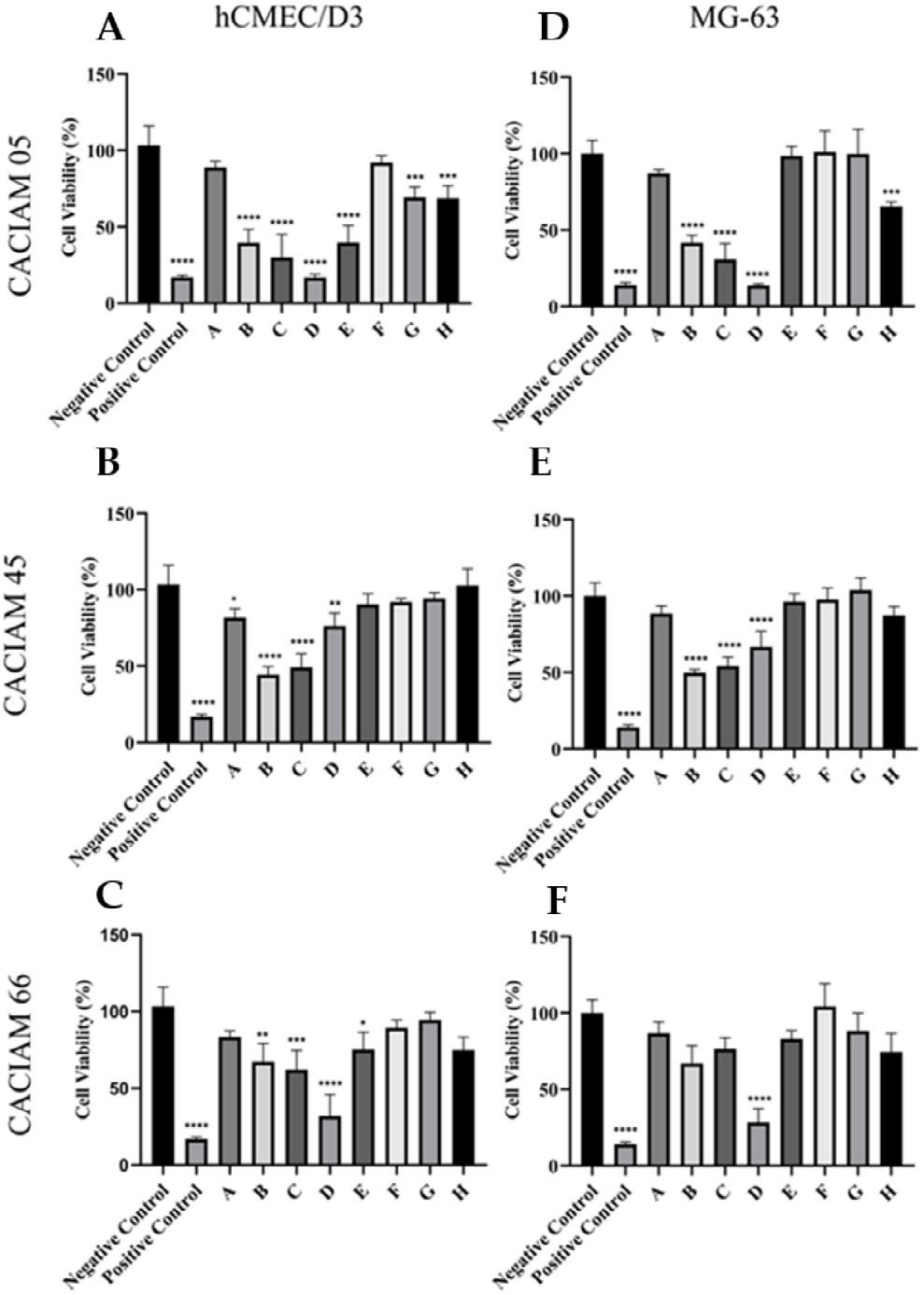
Evaluation of cytotoxicity activity of fractions generated from the MeOH extract of strains CACIAM 05, CACIAM 45, and CACIAM 66 on the cell lines hCMEC/D3 (A–C) and MG‐63 (D–F).

Methanolic (MeOH) extracts derived from CACIAM 05, CACIAM 45, and CACIAM 66 were fractionated for further analysis (Figure 2). The MG‐63 osteosarcoma cell line was utilized as a representative cancer cell model. At a concentration of 25 µg mL^−1^, fraction D from the CACIAM 05 extract (designated as C5FD) exhibited the highest cytotoxicity against both the MG‐63 and hCMEC/D3 cell lines (Figure 2).

### 2.3 Metabolomic Analysis of Fraction D of *Synechocystis* sp. CACIAM 05

The main compounds found in the fraction C5FD showed an *m*/*z* of 623.28487 (Rt: 11.14) and *m*/*z* 653.2931 (Rt: 11.47) (Table 1; Figure S2). Both compounds have no corresponding hit in the GNPS database. The same compounds were detected as majorities in the cytotoxic fraction of the strain *Gloeothece* sp. LEGE 16572, *Limnoraphis robusta* LEGE XX358, and in an unidentified Synechococcales (LEGE 15546) by Ferreira et al. [20]. The authors identified these metabolites through a manual search in the Dictionary of Natural Products and investigation of their MS2 fragmentation pattern as 13‐hydroxy‐phaeophorbide a methyl ester and 15‐hydroxy‐lactone‐pheophorbide an ethyl ester, respectively. In smaller quantities, another pheophorbide with *m*/*z* of 639.29020 was detected in C5FD and identified as 151‐hydroxy‐lactone‐pheophorbide a methyl ester. Notably, the compound was also encountered in the less bioactive fraction C of the same strain, but with a higher intensity. These findings suggest that this metabolite likely contributes little to the cytotoxic activity observed in fraction D.

**TABLE 1:** Annotation of the molecules detected in the bioactive fraction D of the cyanobacterium *Synechocystis* sp. CACIAM 05.

*m*/*z*	Rt (min)	Precursor intensity	Identification	Source
623.2850	11.14	4.51 × 10^10^	13‐Hydroxy‐pheophorbide a methyl ester	DNP
[M+H]^+^			C_36_H_38_N_4_O_6_	
			Δ −1.94 ppm	
639.2797	11.35	1.88 × 10^9^	15‐Hydroxy‐lactone‐pheophorbide a methyl ester	DNP
[M+H]^+^			C_36_H_38_N_4_O_7_	
			Δ −2.54 ppm	
653.2956	11.47	1.60 × 10^10^	15‐Hydroxy‐lactone‐pheophorbide a ethyl ester	DNP
[M+H]^+^				
			C_37_H_40_N_4_O_7_	
			Δ −2.11 ppm	
903.5616	13.65	4.37 × 10^9^	15‐Hydroxy‐lactone‐pheophytin a	DNP
[M+H]^+^			C_55_H_74_N_4_O_7_	
			Δ −1.58 ppm	
887.5669	13.81	1.07 × 10^10^	13‐Hydroxy‐pheophytin a	DNP
[M+H]^+^			C_55_H_74_N_4_O_6_	
			Δ −1.37 ppm	
917.5791	13.93	9.93 × 10^8^	13‐Methyldioxy‐pheophytin a/ficusmicrochlorin B	DNP
[M+H]^+^			C_56_H_76_N_4_O_7_	
			Δ 0.46 ppm	
309.2426	10.51	8.13 × 10^8^	18‐Hydroxy‐9,11,13‐octadecatrienoic acid methyl ester	GNPS
[M+H]^+^			C_19_H_32_O_3_	
			Δ 0.58 ppm	
335.2582	11.29	1.84 × 10^9^	8,9‐Epoxy‐5(*Z*),11(*Z*),14(*Z*)‐eicosatrienoic acid, methyl ester	GNPS
[M+H]^+^			C_21_H_34_O_3_	
			Δ 0.38 ppm	

Pheophorbides and pheophytins, the compound families associated with the antiproliferative property observed in the C5CD fraction, are chlorophyll degradation products known for their anti‐inflammatory, antioxidant, antiviral, and antiproliferative properties. These metabolites can be acquired through the digestion of vegetables and, due to their photosensitivity, have been explored in photodynamic therapy. The anticancer potential of these metabolites has been widely investigated in different cancer cell lines, demonstrating significant inhibitory activity with IC_50_ values around 0.5 µM [22].

UHPLC–HRMS/MS data from the C5FD fraction were pre‐processed using the Mzmine tool and subsequently submitted to the GNPS platform. Parent ions detected in fraction D were used to generate a feature‐based molecular network. Each feature represents an *m*/*z*, retention time, and MS^2^‐spectrum. These features are organized in subnetworks—also referred to as molecular families or clusters—based on the similarity of their associated MS/MS spectra. This approach relies on the principle that structurally related molecules normally exhibit similar fragmentation patterns in MS^2^ analysis. Features that display sufficiently unique MS^2^ spectra and do not group with others are classified as singletons [23].

**FIGURE 3:**
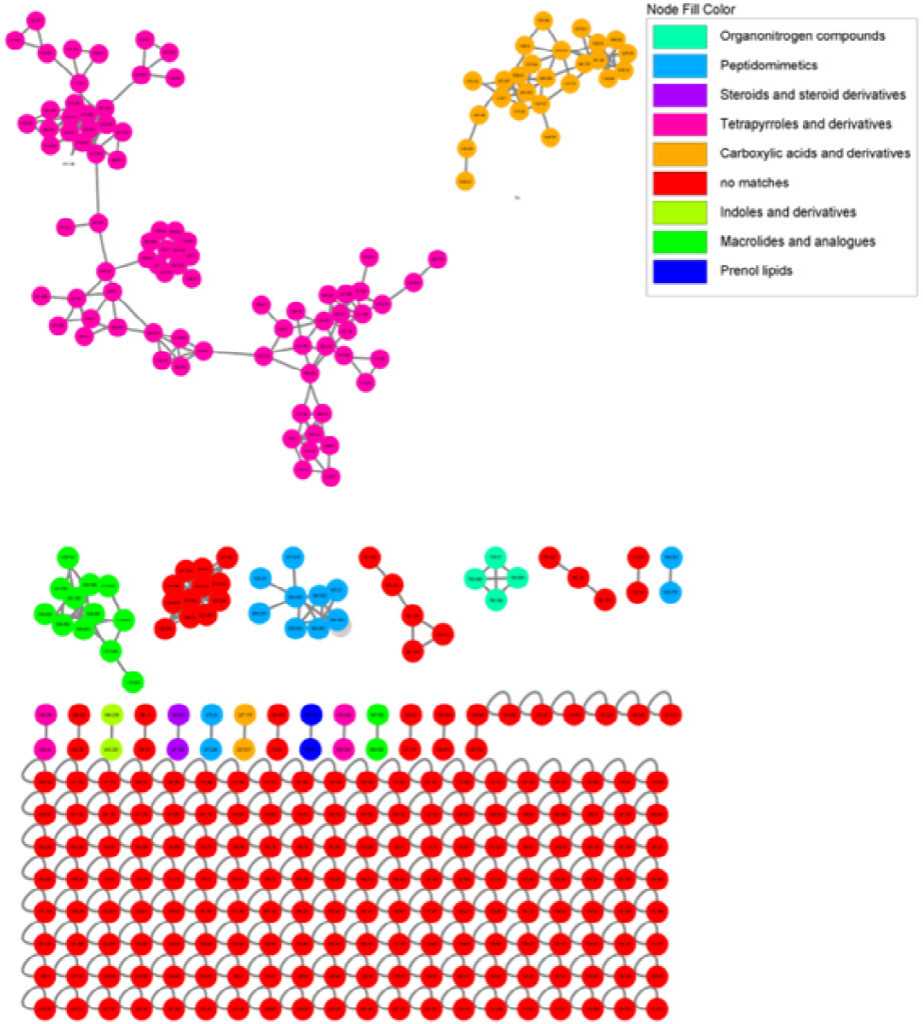
Molecular networking using GNPS constructed from the parent ion present in fraction D of strain *Synechocystis* sp. CACIAM 05. The features highlighted in pink correspond to chlorophyll derivatives.

The chemical composition of the C5FD fraction is mainly characterized by tetrapyrroles and their derivatives (Figure 3), which were responsible for the cytotoxicity activity observed in the fraction. Other chemical groups were identified, such as peptides, macrolides, phenol, and lipids; however, no specific compounds within these groups were identified.

### 2.4 Antimicrobial Potential

Fractions were evaluated for their antimicrobial properties against the Gram‐negative bacterium *Salmonella typhimurium* ATCC 14021, the Gram‐positive *Bacillus subtilis* ATCC 6633, and the yeast *Candida parapsilosis*. None of the fractions investigated were bioactive to the yeast or the Gram‐negative bacterium. The fraction with the highest antibacterial activity was obtained from the strain *Synechocystis* sp. CACIAM 05 (C5FB) (Table 2), followed by fraction C45FC from *Desmonostoc* sp. CACIAM 45 (09.76 ± 0.12 mm). *Synechococcus* sp. CACIAM 66 (C66FD) showed an inhibition halo of 08.68 ± 0.35 mm, comparable to fraction C45FB (08.70 ± 0.23). The results from the agar diffusion assay agreed with those observed in the broth microdilution method. The C5FB fraction displayed a minimum inhibitory concentration (MIC) value of 40 µg mL^−1^ and a minimum bactericide concentration (MBC) value of 80 µg mL^−1^, indicating the occurrence of bactericidal compounds. Fraction C45FC exhibited a MIC value of 80 µg mL^−1^ and an MBC value of 160 µg mL^−1^, while the remaining fractions exhibited low antimicrobial activity with a MIC value superior to 160 µg mL^−1^.

**TABLE 2:** The activity of cyanobacterial fractions against the strain *Bacillus subtilis* ATCC 6633 by disc diffusion assay and determination of the minimum inhibitory concentration (MIC) and minimum bactericidal concentration (MBC) for the bioactive fractions.

Fraction	Inhibitory zone (mm)	MIC (µg mL^−1^)	MBC (µg mL^−1^)
C05FB	12.02 ± 0.07^a^	40	80
C05FC	06.72 ± 0.25^b^	> 160	> 160
C05FF	07.07 ± 0.22^b^	> 160	> 160
C45FB	08.70 ± 0.23^c^	> 160	> 160
C45FC	09.76 ± 0.12^d^	80	160
C66FD	08.68 ± 0.35^c^	> 160	> 160
Tetracycline	25.34 ± 0.06^e^	—	—

The absence of antagonistic activity for Gram‐negative bacteria and yeast documented in this study was also reported by Martins et al. [24], who evaluated the aqueous and organic solvent extracts of several marine cyanobacteria against a panel of Gram‐positive and Gram‐negative bacteria and the yeast *Candida albicans*. None of the extracts tested by the authors exerted inhibitory effects on Gram‐negative bacteria and yeast [24]. These facts can be related to the occurrence of an outer membrane in Gram‐negative bacteria and the chitin cell wall in yeast, which hinders the entrance of toxic substances [25]. The fungal cell wall as a resistance factor was also documented by Humisto et al. [26], who evaluated the mechanism of action of the cyanobacterial peptide hassallidin D. The compound showed deleterious effects on both mammalian cells and yeast, with a very significant difference in half‐maximal effective concentration (EC_50_) values. The reported EC_50_ value for *C. albicans* was 31 ± 3.0 µM, while for mammalian cells it was 4.8 ± 0.56 µM [26].

The production of antimicrobial compounds by cyanobacteria of the order Synechococcales has been reported less frequently than in filamentous ones [27]. In this study, two strains from this group—*Synechococcus* sp. CACIAM 66 and *Synechocystis* sp. CACIAM 05—showed antibacterial activity. Although these microorganisms are known to possess smaller genomes than those of filamentous cyanobacteria, and consequently, a more limited number of biosynthetic gene clusters [28], they may benefit from promiscuity of some of their enzymes, such as the LanM‐type lanthionine synthetase [29]. This bifunctional biocatalyst is vital for the maturation of lanthipeptides. A single LanM enzyme can act on multiple precursor peptides, enabling the production of diverse molecules from the same family within a single strain. In addition, the gene encoding this enzyme has been identified in the genomes of several *Synechococcus* and *Prochlorococcus* species [29].

The fractions from the CACIAM 05 strain demonstrated selectivity toward their targets. The C5FD fraction, for instance, exhibited cytotoxicity against mammary cells, but was unable to inhibit the bacterial growth, even at higher concentrations. Such selectivity may result from the compound's dependence on one or more specific cellular components [30]. For example, the lipopeptide Muscotoxin A, discovered from the soil cyanobacterium *Desmonostoc muscorum*, requires cholesterol to exert its lytic activity, mainly targeting mammalian cells [31]. Despite its high cytotoxicity, the absence of cholesterol in the bacterial membrane likely explains its limited antibacterial activity [31]. A similar cholesterol dependence has been observed in variants of hassallidin D, whose action spectrum excludes Gram‐negative and Gram‐positive bacteria [26].

**FIGURE 4:**
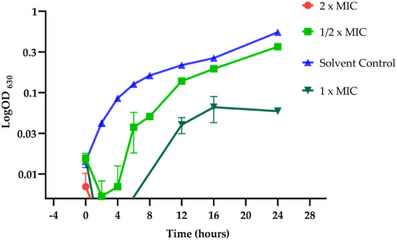
Growth curve of the bacterium *Bacillus subtilis* under different concentrations of the C5FB fraction. As solvent control was utilized, DMSO (4%). Values are expressed as mean ± SD of three determinations (technical replicates).

The bactericidal activity of the fraction C5FB was further evaluated using a bacterial growth curve assay. *B. subtilis* cells, collected from 12‐h culture, were inoculated in Mueller–Hinton (MH) medium at a final concentration of 1 × 10^−5^ CFU mL^−1^. After 2 h of incubation, the cells were exposed to different concentrations of the fractions. As previously observed, the treatment with 80 µg mL^−1^ resulted in complete cell death in less than 1 h (Figure 4). At 40 µg mL^−1^, the fraction exhibited a bacteriostatic effect, with the bacterial growth returning solely after 6 h, reaching maximum absorbance at 16 h. The recorded absorbance (0.06) was approximately nine times lower than that of the control (0.55). A bacteriostatic effect was also documented at 20 µg mL^−1^, although the reduction in growth was less pronounced.

### 2.5 Allelopathic Potential

**FIGURE 5:**
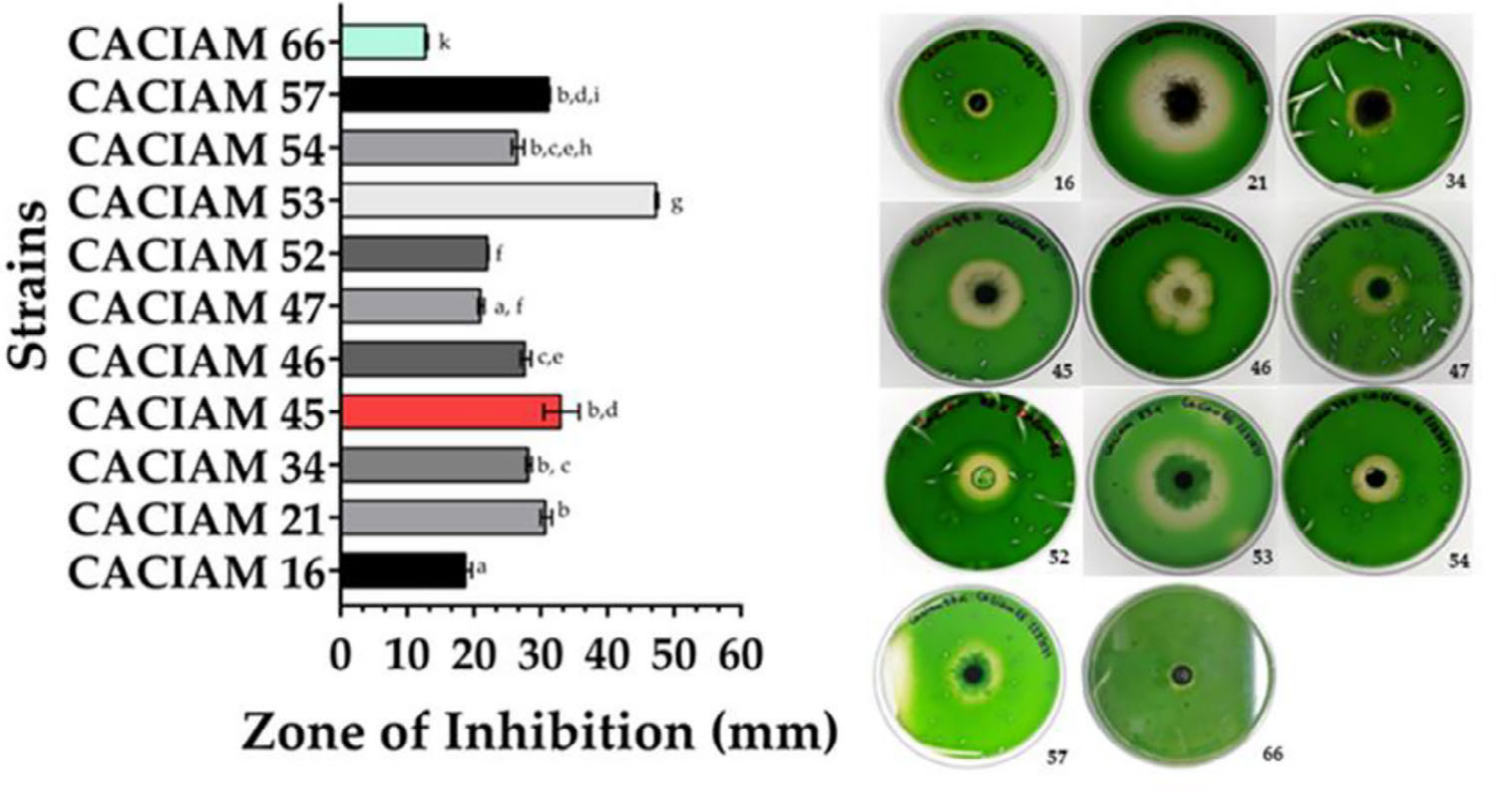
Allelopathic activity of CACIAM strains. Values are expressed as the mean ± SD of three determinations. The strain CACIAM 45 exhibited activity only when tested as a mixed culture. *Synechococcus* sp. CACIAM 66 (in blue) was evaluated against *Microcystis aeruginosa* strain CACIAM 03, while all other strains were tested against CACIAM 66. Different superscript letters in the same row denote statistical differences at *p* ≤ 0.05 (ANOVA, Tukey's HSD).

Among the cyanobacterial strains evaluated for allelopathic potential, filamentous types were identified as the main source of allelochemicals (Table S2). Co‐cultivation in solid media revealed that eleven strains were capable of producing such compounds. Since *Synechocystis* sp. CACIAM 05, *Desmonostoc* sp. CACIAM 45, and *Synechococcus* sp. CACIAM 66 shares the same environmental origin; CACIAM 05 and CACIAM 45 were tested both individually and in combination to investigate a possible synergistic effect. When tested separately, CACIAM 05 and CACIAM 45 showed no inhibitory impact on the growth of CACIAM 66. However, their combination resulted in a significant inhibitory effect, with an inhibition zone of 33.3 mm (Figure 5), suggesting a potential synergistic interaction. The strain CACIAM 53 demonstrated the highest antagonistic potential, with an inhibition zone of 47.43 mm, while *Cyanobium* sp. CACIAM 16 and CACIAM 66 (tested against *Microcystis aeruginosa* CACIAM 03) had the lowest, measuring 18.87 and 12.87 mm, respectively.

The constraints observed between the filamentous and unicellular cyanobacteria during the allelopathic assay can be explained by differences in the genome size between these two groups. Filamentous cyanobacteria typically possess larger genomes than unicellular ones [28]. Hence, they harbor a greater number of biosynthetic gene clusters, which are often associated with the production of allelochemicals. All the cyanobacteria investigated in this study originated from freshwater environments. These cyanobacteria have been recognized as an underexplored yet rich source of algicidal compounds [32]. Among the metabolites discovered from these microorganisms are portoamides extracted from the strain *Oscillatoria* sp. LEGE 05292; fischerellin, identified in the supernatant of the benthic cyanobacterium *Fischerella muscicola* UTEX 1829; hapalindole‐type alkaloids isolated from cyanobacteria of the Stigonematales order, and the calothrixins found in dimethyl sulfoxide (DMSO) extract from *Calothrix* strains [33].

Although the strains CACIAM 45, 05, and 66 are from the same background, it is not possible to confirm that this type of interaction occurs in natural settings. This is due to the high biomass concentration employed in the assay, which is generally found in cyanobacterial blooms and also in most allelopathy studies [34]. However, the present study highlights the potential of some Amazonian strains for bloom control. Microorganisms from this region remain underexplored for their biotechnological potential and are promising candidates for the discovery of novel bioactive compounds [13, 35, 36, 37].

Several studies have demonstrated that exploring the interaction between microorganisms can lead to the discovery of cryptic natural products. These substances are often not produced under standard laboratory conditions or are synthesized in such small quantities that they are difficult to detect [38]. The interaction between CACIAM 05 and CACIAM 45 enabled the production of compounds with algaecide properties, an effect that was not observed when the strains were evaluated individually. A similar phenomenon was reported by Shi et al. [39], who observed the biosynthesis of diphenyl ether by the fungus *Cladosporium* sp. WUH1 as a defensive response to the antifungal cyclopeptides produced by the bacterium *B. subtilis* CMCC(B) 63501. In another study, the strain *M. aeruginosa* FACHB‐905 was found to increase the release of its algaecide compound, linoleic acid, when in contact with the target organism, the green algal *Chlorella vulgaris*. The nitric oxide released by the microalgae stimulated the production of the compound [40].

**FIGURE 6:**
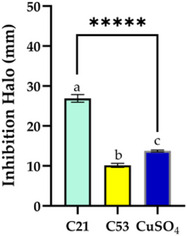
Allelopathic activity of the aqueous (500 µg mL^−1^) and dichloromethane: methanol extracts (1000 µg mL^−1^) of the strains CACIAM 21 (C21) and CACIAM 53 (C53), respectively. Copper sulfate (200 µg mL^−1^) was utilized as the positive control. Values are expressed as the mean ± SD of three determinations. Tukey test was applied to detect significant differences (*p* < 0.0001).

The assay conducted with different types of extracts and concentrations revealed distinct allelopathic behaviors between *Nostoc* sp. CACIAM 21 and CACIAM 53 (Figure 6). For *Nostoc* sp. CACIAM 21, significant inhibitory activity was observed exclusively in the aqueous extract (C21), which produced an inhibition zone of 23.9 ± 0.95 mm at a concentration of 500 µg mL^−1^. This suggests that the active allelopathic compounds in this strain are likely water‐soluble. In contrast, CACIAM 53 exhibited no inhibitory effects at the same concentration (500 µg mL^−1^) for any of its extracts. However, when the concentration was increased to 1000 µg mL^−1^, the dichloromethane–methanol (2:1) extract (C53) displayed an inhibition zone of 10.14 ± 0.51 mm. This indicates that less polar compounds may exert allelopathic effects, but only at higher concentrations. Among the cyanobacteria genus, *Nostoc* is recognized as the most promising due to its production of structurally diverse molecules with various bioactive properties, including algaecides such as nostocine A and nostocyclamide [41].

### 2.6 Total Phenolic Content

The total phenolic content (TPC) of both aqueous and organic extracts was evaluated through the Folin–Ciocalteu method. Table 3 presents the TPC values, expressed in gallic acid (GA) equivalents per milligram of dry extract (µg gallic acid equivalents [GAE] mg^−1^ dry extract), obtained from the strains *Lyngbya* sp. CACIAM 07, CACIAM 45, and CACIAM 66. The highest TPC was observed in the ethanolic extract of strain CACIAM 45 (39.24 ± 1.58 µg GAE mg^−1^ dry extract), followed by its methanolic extract (26.88 ± 0.42 µg GAE mg^−1^ dry extract). With the exception of *Synechococcus* sp. CACIAM 66, the ethanolic extract showed the highest TPC values compared to the methanolic and aqueous extracts (Table 3).

**TABLE 3:** Total phenolic content (µg GAE mg^−1^ dry extract) of cyanobacterial extracts.

Strain	Solvent		
	Aqueous extract	Ethanolic extract	Methanolic extract
*Lyngbya* sp. CACIAM 07	15.43 ± 1.37^a^	22.32 ± 0.12^a^	16.58 ± 0.55^a^
*Desmonostoc* sp. CACIAM 45	17.44 ± 0.52^a^	39.24 ± 1.58^b^	26.88 ± 0.42^b^
*Synechococcus* sp. CACIAM 66	8.94 ± 0.12^b^	6.66 ± 0.86^c^	21.71 ± 0.15^c^

*Note*: Values are expressed as mean ± SD of two determinations. Different superscript letters in the same row denote statistical differences at *p* ≤ 0.05 (ANOVA, Tukey's HSD).

The yield values obtained for the aqueous extract are consistent with those observed by Morone [42, 43] and Favas et al. [44]. The TPC for the aqueous extract reported by these authors ranged from 6.52 ± 0.38 to 15.67 ± 0.23 µg GAE mg^−1^ dry extract, while in our study, they ranged from 8.94 ± 0.12 to 15.43 ± 1.37 µg GAE mg^−1^ dry extract [43, 44]. The differences observed among the extracts can be attributed to the chemical diversity of the various classes of phenolic substances. Cyanobacteria are a rich source of these compounds and exhibit considerable variation in their chemical composition, even at the species level [44]. Ethanol has been widely employed for polyphenol extraction, while methanol is considered suitable for extracting lower molecular weight polyphenols [45]. The yields obtained from biomass using different solvents in the present investigation match those previously reported in another study, where the more polar the solvent, the greater the extraction yield [42, 43, 44].

With respect to the extract yield obtained from dry biomass using different types of solvents, the aqueous extract showed minimal variation among the strains, with yields ranging from 61.15% to 64.85%. Methanolic extract yielded the second‐highest values, whereas ethanolic extract exhibited the lowest yield. This was particularly evident in *Desmonostoc* sp. CACIAM 45, which recorded a yield of only 7.06% (Table 4).

**TABLE 4:** Extract yield using different types of solvents.

Strains	Water	Ethanol	Methanol
*Lyngbya* sp. CACIAM 07	64.85	17.8	20.55
*Desmonostoc* sp. CACIAM 45	61.15	7.06	13.18
*Synechococcus* sp. CACIAM 66	63.7	21.31	26.93

Although extraction with ethanol resulted in a higher phenolic content compared to water and methanol in *Lyngbya* sp. CACIAM 07 and *Desmonostoc* sp. CACIAM 45, the low extraction yields relative to dry biomass provided by the solvent reduced its overall effectiveness. As a result, the phenolic content per gram of dry biomass was 3.97 ± 0.02 and 2.77 ± 0.11 mg GAE g^−1^ for these strains, respectively. These values were considerably lower than those found in the aqueous extract of the same strains, which reached 10.01 ± 0.89 and 10.66 ± 0.32 mg GAE g^−1^ of dry biomass, respectively (Table 5).

**TABLE 5:** Total phenolic content (TPC) of cyanobacterial extracts expressed in mg GAE g^−1^ of dry biomass.

Strain	Solvent		
	Aqueous extract	Ethanolic extract	Methanolic extract
*Lyngbya* sp. CACIAM 07	10.01 ± 0.89^a^	3.97 ± 0.02^a^	3.41 ± 0.11^a^
*Desmonostoc* sp. CACIAM 45	10.66 ± 0.32^a^	2.77 ± 0.11^b^	3.54 ± 0.05^a^
*Synechococcus* sp. CACIAM 66	5.69 ± 0.01^b^	1.42 ± 0.18^c^	5.72 ± 0.04^b^

*Note*: Values are expressed as mean ± SD of two determinations. Different superscript letters in the same row denote statistical differences at *p* ≤ 0.05 (ANOVA, Tukey's HSD).

Similar results were reported by Vega et al. [46], who evaluated the effect of different solvents on the extraction of phenolic compounds. Extraction conducted with water resulted in higher TPC values compared with those performed with ethanol. The *Lyngbya* sp. strain utilized in their study produced an approximate TPC value of 8 mg GAE g^−1^ of dry biomass [46]. These results are aligned with those reported by Morone et al., who evaluated the TPC of cyanobacterial extracts obtained using water and ketone as solvents. While the ketone extract exhibited a higher TPC when measured per gram of extract, the aqueous extract performed better when assessed based on dry biomass weight, primarily due to its greater extraction yield.

**FIGURE 7:**
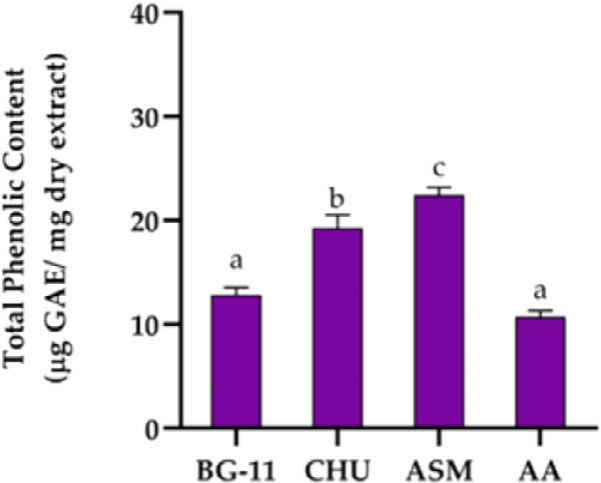
Total phenolic content (µg GAE mg^−1^ dry aqueous extract) determined from CACIAM 45 cells cultivated in BG‐11, CHU‐10, ASM, and AA media. Values are expressed as mean ± SD of three determinations. Different superscript letters in the same bar denote statistical differences at *p* ≤ 0.05 (ANOVA, Tukey's HSD).

The elevated TPC in CACIAM 45 prompted an evaluation of four growth media—BG‐11, ASM, Chu‐10, and AA—to assess their influence on TPC production (Table S3; Figure 7). Among these, ASM resulted in the highest production of phenolic compounds, reaching 22.45 µg GAE mg^−1^ of dry extract, followed by Chu‐10 with 19.28 µg GAE mg^−1^. In contrast, BG‐11 and AA were less effective, with no significant difference between them (*p* ≥ 0.05). These findings identify ASM as the most effective medium for enhancing phenolic compound biosynthesis and antioxidant activity in CACIAM 45.

### 2.7 Determination of PBPs Content

**FIGURE 8:**
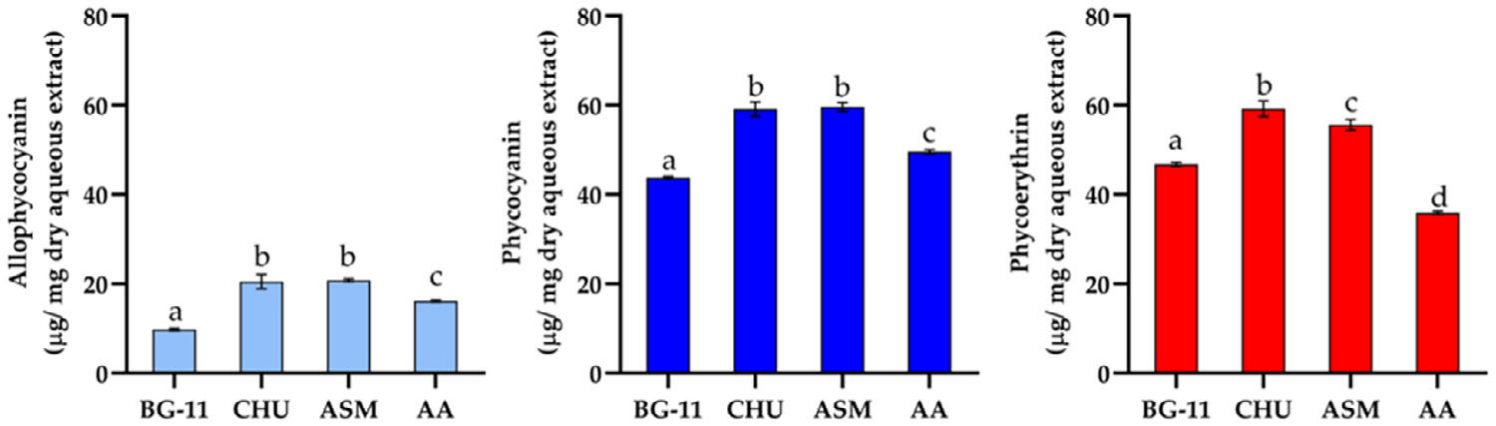
Phycobiliproteins content (µg mg^−1^ dry extract) in CACIAM 45 aqueous extracts grown in different media. Values are expressed as mean ± SD of three determinations. Different superscript letters in the same bar denote statistical differences at *p* ≤ 0.05 (ANOVA, Tukey's HSD).

Other components investigated from the growth of CACIAM 45 under different culture media included PBPs (Figure 8). Over 14 days, the strain adjusted its metabolic processes, leading to alterations in its chemical composition. The culture media employed in this study were selected based on their compositional differences, particularly nitrate and phosphate concentrations. Both elements exert a strong influence on the photosynthetic process and can therefore significantly impact carbon fixation and its allocation into various macromolecule classes [47]. The CHU‐10 and ASM media yielded the highest concentrations of PBPs. Except for phycoerythrin (PE), there were no statistically significant differences between the two media (Figure 8). Both supported the production of very similar concentrations of phycocyanin (PC) (59.09 and 59.60 µg mg^−1^ of dry aqueous extract, respectively) and allophycocyanin (20.52 and 20.76 µg mg^−1^ of dry aqueous extract, respectively). PE concentration showed a slight variation. While CHU‐10 medium yielded 59.19 µg of PE µg mg^−1^ of dry aqueous extract, the growth in the ASM medium resulted in a production of 55.8 µg mg^−1^ of dry aqueous extract.

With the exception of the AA medium, the ratio of PC to PE remained unchanged across the different media, maintaining a proportion near 1:1, indicating that the phycobilisome contains an equal number of PE and PC discs. These variations were visually noticeable in the color of the aqueous extract. The extract from the cells grown in AA medium showed a bluish tint, whereas those collected from the other media showed only slight color differences.

The CHU‐10 and ASM media exhibited the lowest nitrate concentrations compared to the BG‐11 and AA media, and in the present study, provided the most favorable conditions for PBP production. Nitrogen deprivation has been extensively used to enhance the synthesis of secondary metabolites. Certain cyanobacterial strains grown in nitrogen‐free environments, such as *Anabaena* sp. NCCU‐9 and *Anabaena* sp. 7120 showed improved PBP production, as noted by [48] and [49], respectively. However, for some specific strains, removing nitrogen can lead to a significant reduction in both growth and pigment production.

Cyanobacteria normally utilize nitrite, nitrate, and ammonium as nitrogen sources. This element is essential for amino acid biosynthesis, which, in turn, is responsible for protein formation, including the linear tetrapyrrole of PC [50]. Nitrogen deprivation may also lead to the degradation of PBPs since these chromoproteins act as nitrogen reserves, resulting in rapid cell bleaching [51]. In the case of *Desmonostoc* sp. CACIAM 45, the presence of heterocysts and the use of a feast‐and‐famine strategy may help mitigate the stress induced by the absence of nitrogen. This strategy entails growing the strain in a nutrient‐rich environment for a brief period (the “feast”) followed by growth in a more nutrient‐depleted medium (the “famine”) [52].

### 2.8 Antioxidant Activity

Synthetic antioxidants have been extensively employed across various industrial sectors, particularly within the food and cosmetic industries. These compounds are effective in neutralizing free radicals, thereby impeding or slowing the oxidation process. This characteristic has been beneficial in enhancing the flavor, aroma, color, and shelf life of a range of products. However, recent discussions have raised concerns regarding the potential health risks associated with synthetic antioxidants [53]. Consequently, there has been a growing interest in natural antioxidants, which are sourced from organic materials such as plants, microalgae, animals, and bacteria [53]. These natural alternatives not only offer advantages for human health but also hold the potential to promote a circular and eco‐friendly economy by leveraging renewable resources [54].

**FIGURE 9:**
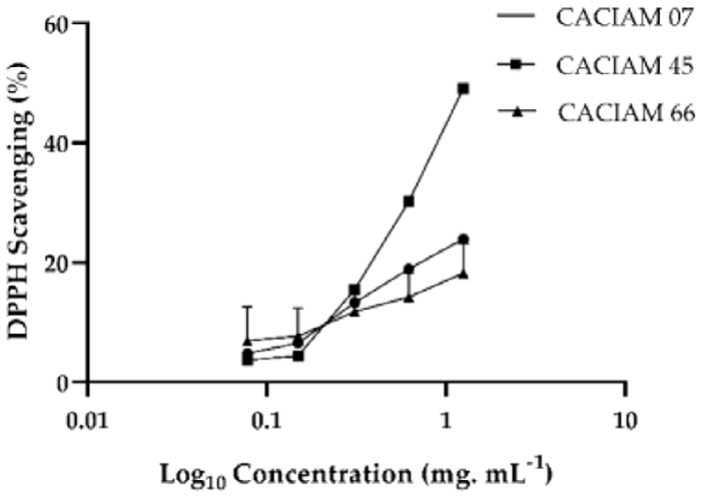
DPPH· scavenging activity of aqueous extracts of *Lyngbya* sp. CACIAM 07, *Desmonostoc* sp. CACIAM 45, and *Synechococcus* sp. CACIAM 66. Values are expressed as mean ± SD of three determinations.

In our study, we evaluated the antioxidant potential of aqueous extracts from CACIAM 07, CACIAM 45, and CACIAM 66 using the 2,2‐diphenyl‐1‐picrylhydrazyl (DPPH) scavenging assay (Figure 9) [55]. This free radical is reduced and neutralized when antioxidant molecules are present, resulting in a colorless or pale‐yellow ethanol solution. Due to its sensibility and low cost, this method has been widely used to investigate the free radical scavenging properties of phenolic compounds and extracts from different sources [55].

CACIAM 45 exhibited the highest DPPH scavenging activity, with an IC_50_ value of approximately 1.25 mg mL^−1^ (Figure 9). The aqueous extract derived from this strain exhibited no cytotoxicity and effectively inhibited microbial growth, while also possessing a high TPC value. These characteristics position this microorganism as a promising candidate for applications in the cosmetic and food industries. In addition, the use of water in the extraction process contributes to reduced solvent costs and minimizes potential risks to human health and the environment.

## 3 Conclusions

This study explored the biotechnological potential of cyanobacteria isolated from the Amazon region. The strains analyzed displayed promising bioactive properties, with particular emphasis on CACIAM 05, which exhibited significant cytotoxic and antimicrobial activity. The observed cytotoxic effect was attributed to the presence of pheophytin and pheophorbide derivatives.

In addition, CACIAM 45 distinguished itself through its significant content of phenolic compounds and PBPs. Furthermore, the extract demonstrates no cytotoxicity, positioning it as a promising candidate for applications in the food and cosmetics industries. Notably, the production of allelochemicals was consistently observed among the cyanobacteria investigated.

In summary, these findings not only expand our understanding of the biotechnological potential of Amazonian cyanobacteria but also highlight the importance of investigating the microbiota within this unique region.

Future research should continue to investigate these organisms in depth to uncover novel metabolic pathways and harness their full potential for innovative biotechnological applications.

## 4 Experimental Section

### 4.1 Culture Strain

**FIGURE 10:**
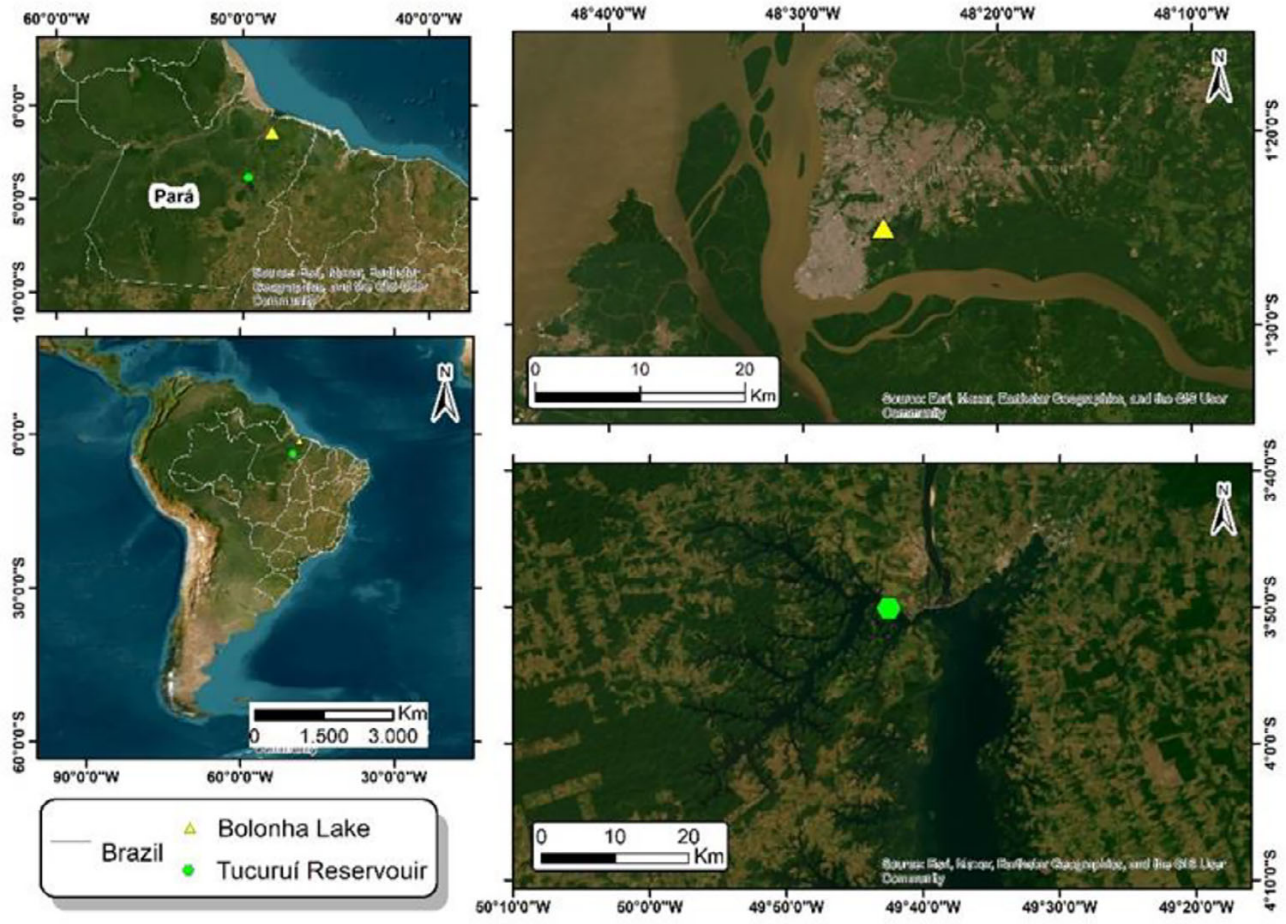
Map displaying the location of the sampling site used to gather environmental samples.

All the cyanobacteria utilized during this study were selected from the Amazonian Collection of Cyanobacteria and Microalgae (CACIAM), which is located in the Biological Institute of the Federal University of Pará (Belém, Pará, Brazil). Table S1 presents general features of these microorganisms. These cyanobacterial strains were recovered from two lakes in the Amazon region: Tucuruí hydroelectric power station reservoir (3°50′04.9″ S, 49°42′32.2″ W) and Bolonha Lake (1°25′00.7″ S, 48°25′52.6″ W), both in Pará, Brazil (Figure 10).

### 4.2 DNA Extraction, Amplification (PCR), and Sequencing

This study identified a cyanobacterium for the first time, initially discovered alongside another cyanobacterium. The strain was purified using the streak plate method and microdilution. After cultivation for 15–20 days in Z8 medium, biomass was harvested, and genomic DNA (gDNA) was extracted with the PureLink Genomic DNA Kit (Invitrogen) using the lysis protocol for Gram‐negative bacteria.

A segment of the 16S rRNA gene was amplified by PCR using the primers 27F and 23S30R. PCR reactions were performed in a final volume of 20 µL containing 1X Green GoTaq Flexi buffer, 2.5 mM MgCl_2_, 125.0 mM of each deoxynucleotide triphosphate, 1.0 µM of each primer, 0.5 U of GoTaq Flexi DNA Polymerase (Promega, Madison, WI, USA), 10 mg mL^−1^ of bovine serum albumin (BSA), and 10–30 ng of template DNA, on a Veriti Dx Thermal Cycler (Thermo Fisher Scientific, Waltham, MA, USA). The PCR conditions were the following: initial denaturation at 94°C for 5 min, followed by 10 cycles of denaturation at 94°C for 45 s, annealing at 57°C for 45 s, and extension at 72°C for 2 min, followed by 25 cycles of denaturation at 92°C for 45 s, annealing at 54°C for 45 s, and extension at 72°C for 2 min with a final elongation step at 72°C for 7 min.

PCR products were separated by 1.5% agarose gel electrophoresis, and DNA fragments of the correct size were purified. Internal primers 359F, 781R, and 1494R were used to improve sequence quality. Sequencing was performed at GATC Biotech, with the sequences assessed and assembled using Geneious software. Potential chimeric artifacts were removed with DECIPHER before phylogenetic analysis [56]. The final sequence was submitted to the BLASTn database for further evaluation. Then it was deposited in the GenBank database (National Center for Biotechnology Information [NCBI], Bethesda, MD, USA) under the accession number PV360734.

### 4.3 Phylogenetic Analysis

The phylogenetic analysis for the taxonomy of CACIAM 45 was conducted using MEGA 12.0. The sequence was aligned with 30 cyanobacterial 16S rRNA sequences from GenBank, using *Synechococcus elongatus* PCC 6301 as the outgroup. A multiple sequence alignment was performed using ClustalW in MEGA 11.0 [57], applying the default gap opening and extension penalties. Evolutionary distances were calculated using the Neighbor‐Joining method with the substitution model GTR+G+I according to the Bayesian information criterion (BIC) and Akaike information criterion (AIC), with 1000 bootstrap replicates conducted to assess the reliability of the branches in the phylogenetic tree. The final phylogenetic tree was revised on iTOL (Interactive Tree of Life) [58].

### 4.4 Culture Conditions and Biomass Harvest

Cyanobacterial strains were inoculated in 4 L of their appropriate growth media (Table S1). Cultures were maintained at 25°C with a photoperiod of 14 h of light and 10 h of darkness, at a light intensity of 10–30 µmol photons m^−2^ s^−1^. Between 21 and 45 days, the unicellular strains were harvested by centrifugation (8000 × *g* for 10 min) while the filamentous strains were collected by filtration using an appropriately sized mesh. The resulting freeze‐dried biomass was then used for extraction.

### 4.5 Biomass Extraction and Fractionation

The freeze‐dried biomass obtained after cultivation was subjected to an extraction process using different solvents: water, methanol, ethanol, and a 2:1 dichloromethane/methanol mixture. For the extraction, the material was macerated, placed in a beaker with the appropriate solvent, and sonicated for five minutes in an ultrasonic water bath at 30°C (Fisher Brand FB15053). Once settled, the supernatant was collected, filtered, and transferred to a round‐bottom flask. This process was repeated four times. The organic extracts were concentrated using a rotary evaporator at 30°C, while the aqueous extract was lyophilized for further analysis.

The crude methanolic extracts of the strain *Synechocystis* sp. CACIAM 05, *Desmonostoc* sp. CACIAM 45, and *Synechococcus* sp. CACIAM 66 was fractionated by reverse phase high‐performance liquid chromatography (HPLC) in a Water Alliance e2695 Separations Module Instrument integrated into a photodiode array detector (Water 2998 PDA) and an automatic Water Fraction Collector III (Waters, Mildford, MA, USA). A volume of 500 µL of the sample at 40 mg mL^−1^ was injected (1 mL loop) and separated on an ACE 10 C8 column (50 × 10 mm, ACE Reading, UK) utilizing a water (H_2_O):acetonitrile (MeCN) gradient (Table 6). Hence, the individual methanolic extract was chromatographed into eight fractions named from A to H. Each fraction exhibits a final volume of 4 mL and was deposited in a 48‐deep well plate (Riplate, Ritter, Schwabmünchen, Germany) [20]. The resulting material was concentrated at 30°C using a rotavapor, weighed, and stored at −20°C.

**TABLE 6:** HPLC chromatographic and collection program for generating fractions.

Time (min)	Flow (mL min^−1^)	MeCN (%)	H_2_O (%)	Collection time (min)	Fraction
0.0	3.0	10	90	1.00–2.30	A
2.0	3.0	80	20	2.30–3.60	B
3.0	3.0	80	20	3.60–4.90	C
4.0	3.0	100	0	4.90–6.20	D
8.9	3.0	100	0	6.20–7.50	E
9.2	3.5	100	0	7.50–8.80	F
12.0	3.5	100	0	8.80–10.36	G
12.3	3.0	100	0	10.36–11.50	H
14.0	3.0	100	0		
15.0	3.0	10	90		
18.0	3.0	10	90		

### 4.6 Cytotoxicity Assay

#### 4.6.1 Cell Culture

For the cytotoxicity assay, the employed human brain endothelial cell line hCMEC/D3 was generously donated by Dr. P. O. Courad (INSERM, Paris, France). The cancerous cell lines employed in this study included the human colon carcinoma cell line HCT‐116, the human hepatoma‐derived cell line HepG2, and the human osteosarcoma cell line MG‐63, all sourced from Sigma‐Aldrich (St. Louis, MO, USA). The HCT‐116 cell line was cultured in McCoy's 5A medium (CarlRoth, Karlsruhe, Germany), while hCMEC/D3, HepG2, and MG‐63 were maintained in Dulbecco's modified Eagle medium (DMEM) (Gibco, Thermo Fisher Scientific). Both media were supplemented with 10% fetal bovine serum (Biochrom, Berlin, Germany), 1% penicillin/streptomycin (Biochrom, Berlin, Germany), and 0.1% amphotericin (GE Healthcare, Little Chalfont, Buckinghamshire, UK). All cell lines were incubated in a humidified atmosphere containing 5% CO_2_ at a temperature of 37°C.

#### 4.6.2 Bioactivity Screening

The cytotoxicity of the fractions and extracts was assessed using the MTT colorimetric assay. This assay measures the reduction of yellow MTT dye to insoluble purple formazan crystals by the enzyme succinate dehydrogenase in mitochondria of viable cells. After the cell growth, the medium was removed and the cells were washed between two to three times with phosphate‐buffered saline solution pH 7.4 (Gibco, Thermo Fisher Scientific) and then detached from the flask using the TrypLE solution (Gibco, Thermo Fisher Scientific). Subsequently, the cells were seeded on 96‐well plates at a density of 3.3 × 10^4^ cells mL^−1^ for 24 h. Both extracts and fractions were subsequently evaluated. Methanolic and aqueous extracts were analyzed at final concentrations of 200–12.5 µg mL^−1^, while fractions were tested at 25 µg mL^−1^. We used 0.5% DMSO as the vehicle control, serving as a reference for 100% cell viability, and 20% DMSO as the positive cytotoxicity control [42, 44].

After 24 and 48 h of exposure for the extracts and 48 h for the fractions, the cells were incubated with 20 µL of MTT reagent (200 µg mL^−1^) for 4 h as previously described by [20, 42]. Following this, the supernatant was removed, and 100 µL of DMSO was added to dissolve the formazan crystals. Absorbance was measured at 550 nm using a Synergy HT Multi‐Detection Microplate Reader (BioTek, Bad Friedrichshall, Germany), operated with GEN5 software. All assays were performed in triplicate. Cell viability was quantified using the specified formula:

$$\begin{array}{ccc} & & \%\;\mathrm{cell}\;\mathrm{viability}\;\left(\mathrm{to}\;\mathrm{negative}\;\mathrm{control}\right)\\ & & \;=\frac{x\;\left(\mathrm{Absorbance}\;\mathrm{sample}\right)}{x\;\left(\mathrm{Absorbance}\;\mathrm{negative}\;\mathrm{control}\right)}\times 100 \end{array}$$

### 4.7 Metabolite Profiling

The most cytotoxic fraction (C5FD) was analyzed on a Q Exactive Focus mass spectrometer equipped with an electrospray ionization source and coupled to Dionex UltiMate 3000 UHPLC with an MWD‐3000RS UV/VIS detector (Thermo Fisher Scientific). The fraction was resuspended in LC–MS grade MeOH to a final concentration of 1 mg mL^−1^ and subsequently was filtered using a 0.2 µm syringe filter. HPLC separation was performed with 5 µL of sample volume with a flow rate of 0.35 mL min^−1^ on an ACE Ultracore 2.5 SuperC18 column (75 × 2.1 mm, ACE, Reading, UK) using a gradient from 99.5% to 10% of Mobile Phase A (95% H_2_O, 5% MeOH, 0.1% v/v formic acid) and 0.5% to 90% of Mobile Phase B (95% isopropanol, 5% MeOH, 0.1% v/v formic acid) for 9.5 min. The last proportion (A:B 10:90) was maintained for six minutes, with a total running time of 15.5 min before returning to the initial conditions. The temperature of the column oven was set to 40°C, and the UV absorbance of the eluate was monitored at 254 nm. The liquid chromatography tandem to a high‐resolution electrospray ionization mass spectrometry (LC–HRESIMS/MS) analysis was acquired in the positive mode using a spray voltage of 3.5 kV with a capillary temperature maintained at 262.5°C. Also, a full MS scan was performed at the resolution of 70 000 FWHM (*m*/*z* range of 150‐2000) as well as the data‐dependent MS^2^ discovery mode (ddMS2 discovery acquisition mode) at the resolution of 17 500 FWHM (isolation window used was 3.0 amu and normalized collision energy was 35). Raw data were converted to the open format. mzML using the MSCconvert software according to the parameters recommended for the Global Natural Product Social Molecular Networking (GNPS). Mzmine 2 v.2.53 (https://github.com/mzmine/mzmine2/releases) was employed to generate a quantification file, which was utilized to determine the ion precursor intensity. The parameters utilized were the same as those presented by [20]. Data obtained from the analyses with the GNPS tools DEREPLICATION, MS2LDA, and Network Annotation Propagation (NAP) were combined using the MolNetEnhancer workflow [59]. The resulting molecular network was visualized through Cytoscape 3.8.3 software. The Dictionary of Natural Products Chemical Search (https://dnp.chemnetbase.com) and CyanoMetDB [7] were utilized in the manual dereplication of the metabolites.

### 4.8 Antimicrobial Assay

#### 4.8.1 Determination of Antimicrobial Activity Using the Agar Disc Diffusion Method

The bacteria *B. subtilis* ATCC 6633 and *S. typhimurium* ATCC 14021 were inoculated in 5 mL of MH medium and then incubated at 37°C for 24 h. The antibacterial property of the cyanobacterial fractions was evaluated using the standard agar disc diffusion method with slight modifications [60]. The volume of 100 µL of diluted bacterial cultures (DO_630_: 0.12) was seeded on the surface of MH agar (1.5 %) medium. Sterile filter paper discs (6 mm) were impregnated with 10 µL of the fraction at 2 mg mL^−1^ dissolved in DMSO. DMSO was employed as the negative control. The plates were incubated at 37°C for 24 h and the size of the zone of inhibition was measured in mm using a digital pachymeter. The yeast *C. parapsilosis* was cultivated in 5 mL of Sabouraud medium for 72 h at 35°C. The cells were harvested by centrifugation (10 min, 4000 × *g*) and then resuspended at a final DO_630_: 0.3. An aliquot of 10 µL was placed and spread on the surface of a Sabouraud dextrose agar medium plate (HiMedia). Paper disks were prepared and added in the same way as with bacteria. Then, the plates were incubated at 35°C for 48 h. Disks of tetracycline were used as a positive control.

#### 4.8.2 Determination of MIC and MBC Values

The MIC for those microorganisms with the sensibility to bioactive cyanobacterial fractions was determined by the broth microdilution method in MH medium for the bacteria *B. subtilis* in a 96‐well plate as reported by Clinical and Laboratory Standards Institute Performance Standard for Antimicrobial Susceptibility Testing M100 with few modifications [60]. Briefly, the wells from the first column were all utilized as negative growth control, being filled only with 100 µL of the growth medium. In the wells from the second to the eighth column were added 96 µL of the bacterial culture with a final cell concentration of 5 × 10^5^ CFU mL^−1^. Subsequently, 4 µL of fractions were added to a final concentration of 160, 80, 40, 20, 10, and 5 µg mL^−1^. Columns 9 and 10 were utilized to test the negative and the positive control using DMSO (4%) and chloramphenicol (50 µM), respectively. The MIC was defined as the lowest fraction concentration with no visible growth after 24 h of incubation. The MBC was determined following the MIC characterization. Ten microliters of samples from the wells without visible growth were spread on MH agar medium and then incubated at 37°C for 48 h. MBC was registered as the lowest concentration, where growth was not detected. The assay was performed in triplicate and averaged.

#### 4.8.3 Microbial Growth Curve Assay

The influence of various concentrations of the C5FB fraction on the growth of *B. subtilis* ATCC 6633 was evaluated by constructing a growth curve. The bacterium was cultured in MH broth at 37°C for 12 h, then centrifuged and resuspended to a final concentration of 1 × 10^5^ CFU mL^−1^. After a 2‐h incubation, the cells were exposed to C5FB fractions at 0.5 × MIC, MIC, and 2 × MIC, with a control group maintained using 4% DMSO. Growth was monitored by measuring optical density at 630 nm over 24 h.

### 4.9 Evaluation of Allelopathic Activity

#### 4.9.1 Cocultivation in Solid Medium

The allelopathic potential of the cyanobacteria was evaluated through co‐cultivation in a solid medium assay [61]. As target organisms were employed the unicellular strains *Synechococcus* sp. CACIAM 66 and *M. aeruginosa* CACIAM 03. The cyanobacteria *Desmonostoc* sp. CACIAM 45 and *Synechocystis* sp. CACIAM 05 were tested as a mixture and separately. Cells in the exponential growth phase of the target cyanobacteria were harvested by centrifugation at 10 000 × *g* for 15 min at 21°C and then added to BG‐11‐agar media (1.5%) to a final absorbance of 0.65 at 750 nm. The plates were maintained at 25°C with 14 h of light and 10 h of darkness for 48 h. After this period, a circle of agar (7 mm) was removed from the middle of the plate and filled with 200 µL of the test cyanobacterium (0.4 g of fresh biomass mL^−1^) previously mixed with BG‐11‐agar (0.4%). The plates were thus incubated for 21 days or until the inhibition halo formation, which was measured using a digital pachymeter.

#### 4.9.2 Evaluation of the Extract Effect on the *Synechococcus* sp. CACIAM 66 Growth

In the center of CACIAM 66‐containing plates, an agar disk was excised and replaced with 100 µL of aqueous and organic extract obtained from the filamentous cyanobacteria *Nostoc* sp. CACIAM 21 and CACIAM 53. The extracts were tested at final concentrations of 500 and 1000 µg mL^−1^, respectively. Copper sulfate was utilized as the positive control at a concentration of 200 µg mL^−1^, while DMSO served as the negative control. The inhibition halos were measured after 1 week using a digital pachymeter. The experiment was conducted in triplicate to ensure reliability, and the results were subsequently averaged for analysis.

### 4.10 Evaluate of TPC of the Extracts

The TPC values of the extract of the cyanobacteria were determined utilizing the colorimetric method of Folin–Ciocalteu [42], which is a solution capable of reacting with the reduced phenols and producing a stable blue product at the end of the reaction. The reaction mixture was composed of 25 µL of extract (10 mg mL^−1^) combined with 25 µL of Folin–Ciocalteu reagent (Sigma‐Aldrich), 200 µL of Na_2_CO_3_ (75 mg mL^−1^), and 500 µL of deionized water. The solution was thoroughly mixed and then 200 µL was transferred in duplicate to a 96‐well plate, which was kept in total darkness for 1 h at room temperature. After this period, the absorbance was measured at 725 nm utilizing a Cytation Hybrid Multimode Reader (BioTek Instruments, Winooski, VT, USA). Two standard calibration curves were constructed with seven concentrations of gallic acid (GA) (0.025 to 0.5 mg mL^−1^). One using DMSO as solvent (Abs = 2.0542 [GA] + 0.0914, *R* ^2^ = 0.9968) for the evaluation of methanolic and ethanolic extracts and another utilizing water for the analysis of the aqueous extract (Abs = 2.3076 [GA] + 0.102, *R* ^2^ = 0.9916). Total phenols in each extract were expressed in mg GAE g^−1^ dry biomass. The experiment was carried out in duplicate and averaged. For reproducibility, each assay was independently repeated twice.

### 4.11 Determination of PBPs Content

The aqueous extract of the strain *Desmonostoc* sp. CACIAM 45, sourced from four distinct media—BG‐11 [62], Chu‐10 [63], Allen and Arnon (AA), and ASM [64]—was evaluated for its phenolic compounds and PBPs content. The PBPs were quantified spectrophotometrically by measuring the optical density at multiple wavelengths (562, 615, and 645 nm) using a cell with a 1 cm optical path. The relevant equations applied in this analysis were adapted from the methodologies outlined by Pagels et al. [65] (below).

$$\mathrm{Phycocyanin}\;\left(\mathrm{PC}\right)=\frac{A_{615\mathrm{nm}}-0.474\times A_{652\mathrm{nm}}}{5.34}$$

$$\mathrm{Allophycocyanin}\;\left(\mathrm{APC}\right)=\frac{A_{652\mathrm{nm}}-0.208\times A_{615\mathrm{nm}}}{5.09}$$

$$\mathrm{Phycoerythrin}\;\left(\mathrm{PE}\right)=\frac{A_{562\mathrm{nm}}-2.41\times \mathrm{PC}-0.849\times \mathrm{APC}}{9.62}$$

### 4.12 DPPH• Scavenging Activity

DPPH free radical scavenging activity of the aqueous extract from *Lyngbya* sp. CACIAM 07, *Desmonostoc* sp. CACIAM 45, and *Synechococcus* sp. CACIAM 66 was determined using the method described by Xie and Schaich [66] with some alterations. Briefly, a volume of 25 µL of the aqueous extract was mixed with 200 µL of DPPH• reagent (100 µM) freshly prepared in methanol to final concentrations of 1, 0.5, 0.25, 0.13, 0.07 mg mL^−1^ in a 96‐well plate. DMSO was used as a negative control, and GA as a positive control. The absorbance (Abs) was measured at 515 nm after 15 min of incubation at ambient temperature in the dark using a Synergy HT Multi‐detection Microplate Reader operated with GEN5TM (Biotek). The assay was performed in triplicate. Results were expressed as a percentage of radical scavenging activity compared to the untreated control.

### 4.13 Statistical Analysis

With the exception of the TPC determination, all experiments were performed in triplicate and expressed as mean ± standard deviation. Analysis of variance was conducted by Dunnett's test for the cytotoxicity assay and Tukey's test for the others, following one‐way ANOVA; differences at *p* < 0.05 were considered statistically significant, using the software GraphPad 8.0 (GraphPad Software, San Diego, CA, USA, https://www.graphpad.com/).

## Author Contributions

**Samuel do Amaral**: conceptualization, methodology, investigation, data curation, and writing – original draft preparation, review and editing. **Luciana Xavier**: conceptualization, methodology, writing – review and editing, data curation, resources, and visualization. **Mariana Reis**: conceptualization, methodology, data curation, visualization, and supervision. **Raquel Silva**: methodology, visualization, and writing – review and editing. **Rhuana V. Médice**: data curation, and writing – review and editing. **Janaina Morone**: methodology, data curation, and writing – review and editing. **João Morais**: methodology. **Vitor Vasconcelos**: conceptualization, data curation, writing – review and editing, supervision, and funding acquisition. **Agenor Valadares**: conceptualization, data curation, writing – review and editing, supervision, funding acquisition.

## Conflicts of Interest

The authors declare no conflicts of interest.

## Supporting information




**Supporting File 1**: cbdv70511‐sup‐0001‐SuppMat.docx

## Data Availability

The data that support the findings of this study are available from the corresponding author upon reasonable request.
