# Supplementary material for: Amazonian Cyanobacteria as a Source of Bioactive Compounds With Antimicrobial and Cytotoxic Properties
Source: Chem Biodivers. 2025 Sep 22;22(12):e01582. doi: 10.1002/cbdv.202501582 (PMC12715989; doi:10.1002/cbdv.202501582)
Supplement: Supplementary file 1 — Supporting File 1: cbdv70511‐sup‐0001‐SuppMat.docx [file CBDV-22-e01582-s001.docx]

**Supplementary Material**

**Amazonian Cyanobacteria as a Source of Bioactive Compounds With Antimicrobial and Cytotoxic Properties**

Samuel Cavalcante do Amaral*^1^, Luciana Pereira Xavier^1^, Mariana Reis^2^, Rhuana V Médice^3^, Janaína Morone^2,4^, Raquel Silva^2^, João Morais^2^, Vitor Vasconcelos^2,4^, and Agenor Valadares Santos*^1^.

^1^Laboratory of Biotechnology of Enzymes and Biotransformation, Biological Sciences Institute, Federal University of Pará, Belém 66075-110, Brazil ([samuel.amaral@usp.br](mailto:samuel.amaral@usp.br); [lpxavier@ufpa.br](mailto:lpxavier@ufpa.br); [avsantos@ufpa.br](mailto:avsantos@ufpa.br)).

^2^CIIMAR/CIMAR-LA—Interdisciplinary Centre of Marine and Environmental Research, University of Porto, Terminal de Cruzeiros do Porto de Leixões, Av. General Norton de Matos s/n, 4450-208 Matosinhos, Portugal ([mreis@ciimar.up.pt](mailto:mreis@ciimar.up.pt); [janabavini@ciimar.up.pt](mailto:janabavini@ciimar.up.pt); [rssilva@ciimar.up.pt](mailto:rssilva@ciimar.up.pt); [jmorais@ciimar.up.pt](mailto:jmorais@ciimar.up.pt); [vmvascon@fc.up.pt](mailto:vmvascon@fc.up.pt)).

^3^Department of Clinical Chemistry, School of Pharmaceutical Sciences, University of São Paulo, Av. Professor Lineu Prestes, 580 - Bloco 17, São Paulo, SP, 05508-000, Brazil ([medicerv@usp.br](mailto:medicerv@usp.br)).

^4^Departamento de Biologia, Faculdade de Ciências, Universidade do Porto, Rua do Campo Alegre, Edifício FC4, 4169-007 Porto, Portugal

|  |
| --- |

**Table S1**. Strains utilized in this study

| **Strain** | **Morphology** | **Source** | **Assay** | **Media** |
| --- | --- | --- | --- | --- |
| *Microcystis* sp.  CACIAM 03 | Unicellular | Freshwater | Allelopathy | BG-11 |
| *Microcystis* sp.  CACIAM 04 | Unicellular | Freshwater | Allelopathy | BG-11 |
| *Synechocystis* sp. CACIAM 05 | Unicellular | Freshwater | Antimicrobial  Cytotoxicity  Allelopathy | Z8 and BG-11 |
| *Lyngbya* sp.  CACIAM 07 | Filamentous | Freshwater | Antioxidant  Phenolic Contents  Allelopathy | Z8 |
| *Microcystis* sp.  CACIAM 08 | Unicellular | Freshwater | Allelopathy | BG-11 |
| *Cyanobium* sp.  CACIAM 16 | Unicellular | Freshwater | Allelopathy | BG-11 |
| Nostoc sp.  CACIAM 19 | Filamentous | Freshwater | Allelopathy | BG-11 |
| *Nostoc* sp.  CACIAM 21 | Filamentous | Freshwater | Allelopathy | BG-11 |
| CACIAM 28 | Unicellular | Freshwater | Allelopathy | BG-11 |
| CACIAM 29 | Unicellular | Freshwater | Allelopathy | BG-11 |
| CACIAM 34 | Filamentous | Freshwater | Allelopathy | BG-11 |
| *Desmonostoc* sp. CACIAM 45 | Filamentous | Freshwater | Cytotoxicity  Antimicrobial  Allelopathy  Phenolic Contents  Antioxidant  Phycobiliprotein | Z8, BG-11, CHU-10, ASM, AA |
| CACIAM 46 | Filamentous | Freshwater | Allelopathy | BG-11 |
| CACIAM 47 | Filamentous | Terrestrial | Allelopathy | BG-11 |
| CACIAM 52 | Filamentous | Freshwater | Allelopathy | BG-11 |
| CACIAM 53 | Filamentous | Freshwater | Allelopathy | BG-11 |
| CACIAM 54 | Filamentous | Freshwater | Allelopathy | BG-11 |
| CACIAM 57 | Filamentous | Freshwater | Allelopathy | BG-11 |
| *Synechococcus* sp. CACIAM 66 | Unicellular | Freshwater | Cytotoxicity  Antimicrobial  Allelopathy | Z8 and BG-11 |

**Table S2**. Details of forward (1 -2) and reverse (3-5) primers used in this study to amplify the 16S rRNA gene of cyanobacteria

| **Number** | **Primer Name** | **Sequence (5’-3’)** |
| --- | --- | --- |
| 1 | 27F | AGAGTTTGATCCTGGCTCAG |
| 2 | 359F | GGGGAATYTTCCGCAATGGG |
| 3 | 781R | GACTACWGGGGTATCTAATCCCWTT |
| 4 | 1494R | TACGGCTACCTTGTTACGAC |
| 5 | 23S30R | CTTCGCCTCTGTGTGCCTAGGT |


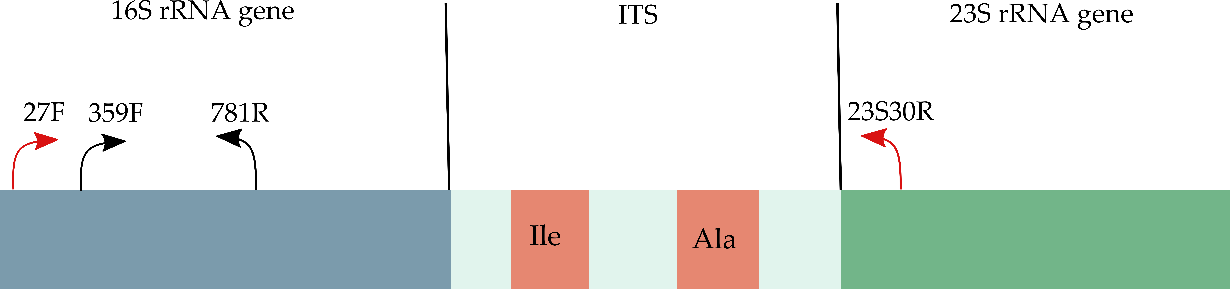


**Figure S1**. Schematic diagram representing the primer pairs utilized in this study. The primer set 27F and 23S30R (red) was employed to amplify the 16S-ITS-23S gene while the primer pair 359F a and 781R (black) was used to amplify the cyanobacterial 16S rRNA gene.

**Figure S2.** Total ion chromatogram of fraction D obtained from the methanolic extract of Synechocystis sp. CACIAM 05

**Table S3.** Constituents of the media used for optimizing the production of phenolic compounds and phycobiliproteins in the Desmonostoc sp. CACIAM 45

|  | **Final Concentration (g/L)** | | | |
| --- | --- | --- | --- | --- |
| **Component** | **BG-11** | **ASM I** | **Chu-10** | **AA** |
| **Na_2_EDTA** | 0.001 | 0.00744 |  | 0.004 |
| **Citric acid** | 0.006 |  | 0.003 |  |
| **NaNO_3_** | 1.5 | 0.17 |  |  |
| **KNO_3_** |  |  |  | 2.02 |
| **Ca (NO_3_)_2_.4H_2_O** |  |  | 0.05 |  |
| **K_2_HPO_4_.3H_2_O** | 0.04 | 0.00174 | 0.013 | 0.456 |
| **MgSO_4_.7H_2_O** | 0.075 | 0.049 | 0.025 | 0.456 |
| **CaCl_2_.2H_2_O** | 0.036 | 0.026 |  | 0.074 |
| **NaCl** |  |  |  | 0.232 |
| **Na_2_CO_3_ (H_2_O)** | 0.02 |  | 0.02 |  |
| **FeCl_3_** |  | 0.00108 | 0.003 |  |
| **MgCl_2_.6H_2_O** |  | 0.041 |  |  |
| **Na_2_HPO_4_.7H_2_O** |  | 0.00266 |  |  |
| **H_3_BO_3_** |  | 0.00248 |  |  |
| **MnCl_2_.4H_2_O** |  | 0.00139 |  |  |
| **C_6_H_8_FeNO_7_** | 0.006 |  |  |  |
| **ZnCl_2_** |  | 0.00034 |  |  |
| **CoCl_2_.6H_2_O** |  | 1.9E-05 |  |  |
| **CuCl_2_** |  | 1.4E-06 |  |  |
| **Micronutrients** | 1 mL |  |  |  |
|  |  |  |  |  |

**Table S4**. Composition of the BG-11 medium micronutrient solution.

| **Components** | **Stock Solution Concentration (g/L)** |
| --- | --- |
| H_3_BO_3_ | 2.86 |
| MnCl_2_.4H_2_O | 1.81 |
| ZnSO_4_.7H_2_O | 0.222 |
| Na_2_Mo_4_.2H_2_O | 0.39 |
| CuSO_4_.5H_2_O | 0.079 |
| Co(NO3)_2_.6H_2_O | 0.049 |
